# Serious Mental Illness and Dementia in Assisted Living

**DOI:** 10.1093/geroni/igaa057.2353

**Published:** 2020-12-16

**Authors:** Cassandra Hua, Portia Cornell, Kali Thomas

**Affiliations:** 1 Brown University School of Public Health, Providence, Rhode Island, United States; 2 Providence VA Medical Center, Providence, Rhode Island, United States; 3 Brown University, Providence, Rhode Island, United States

## Abstract

Little is known about trends in the prevalence of serious mental illness (SMI) and Alzheimer’s disease and Related Dementias (ADRD) in assisted living (AL). We summarize changes in the prevalence of SMI and ADRD in larger AL settings (25+ beds) from 2008-2017 using Medicare claims data. We compare these changes to nursing home (NH) and community rates of SMI and ADRD. We also examine state variability in SMI and ADRD in AL in 2017. The prevalence of SMI in AL increased 37%, from 7.8% in 2008 to 10.7% in 2017; ADRD prevalence increased 34%, from 27% to 36.4%. Over time, NHs exhibited the greatest increases in SMI (53%), followed by AL (37%) and the community cohorts (27%). Increases in ADRD were highest in AL. Rates of SMI in AL ranged from 3.5% in Wyoming to 28.7% in New York. We discuss implications for future research and policy.

